# Salvage Surgery: A Concrete Opportunity in Unresectable Non-Small Cell Lung Cancer Following Definitive Chemo-Immunotherapy †

**DOI:** 10.3390/cancers17182967

**Published:** 2025-09-10

**Authors:** Maria Giovanna Mastromarino, Elena Guerrini, Lisa Maria Caciagli, Andrea La Rosa, Diana Bacchin, Vittorio Aprile, Stylianos Korasidis, Alessandra Lenzini, Alessandra Celi, Greta Alì, Marcello Carlo Ambrogi, Marco Lucchi

**Affiliations:** 1 Division of Thoracic Surgery, Cardiac, Thoracic and Vascular Department, University Hospital of Pisa, 56124 Pisa, Italy; 2 Department of Surgical, Medical and Molecular Pathology and Critical Care Medicine, University of Pisa, 56124 Pisa, Italy; 3 Unit of Pathological Anatomy, Department of Surgical, Medical and Molecular Pathology and Critical Care Medicine, University Hospital of Pisa, 56124 Pisa, Italy

**Keywords:** salvage surgery, non-small cell lung cancer, immune checkpoint inhibitors, neoadjuvant therapy, chemo-immunotherapy, complete pathologic response, major pathologic response

## Abstract

Background: The advent of immunotherapy has significantly improved survival outcomes in advanced non-small cell lung cancer (NSCLC). In this evolving context, salvage surgery has emerged as a potential curative strategy, despite the risk of serious complications. This study aimed to evaluate the safety and efficacy of surgical resection following chemo-immunotherapy in patients with initially unresectable NSCLC. Methods: We retrospectively analyzed patients with stage III–IVB NSCLC who underwent salvage surgery at our institution between January 2019 and June 2024. All cases were initially deemed unresectable by a multidisciplinary tumor board. Perioperative complications, complete (R0) resection rate, major pathologic response (MPR), complete pathologic response (pCR), progression-free survival (PFS), and overall survival (OS) were analyzed. Results: Twenty-one patients (thirteen males, eight females; median age: 68 years [IQR: 9]) were included. Reasons for initial unresectability were metastatic disease (28.6%), N2 bulky disease (14.3%), local invasiveness (33.3%), or a combination of factors (23.7%). Chemo-immunotherapy was administered in 19 patients (90.5%), while 2 (9.5%) received immunotherapy alone, with a median of four treatment cycles (IQR: 1). Complete (R0) resection was achieved in all patients (100%). Anatomical resections were performed in 17 patients (81%), predominantly lobectomies (66.7%). There were no intraoperative or major postoperative complications, and 30-day mortality was zero. Median hospital stay was 7 days (IQR: 4). pCR and MPR were achieved in 33.3% and 14.3% of patients, respectively. After a median follow-up of 17 months (IQR: 19), the estimated 3-year PFS and OS were 50.9% and 66.3%, respectively. Recurrences included locoregional (4.8%), distant (14.3%), and combined (14.3%). Cox regression analysis identified stage III at diagnosis (OR: 0.292; 95% CI: 0.093–0.912; *p* = 0.034) and achieved pCR or MPR (OR: 0.113; 95% CI: 0.013–0.959; *p* = 0.046) as independent predictors of improved PFS. Conclusions: Salvage surgery after chemo-immunotherapy in initially unresectable NSCLC appears to be a safe and effective strategy in selected patients, offering favorable pathological responses and encouraging mid-term oncologic outcomes.

## Simple Summary

Our study highlights the potential of salvage surgery as a curative option in patients with initially unresectable non-small cell lung cancer (NSCLC) who achieve significant clinical response following definitive chemo-immunotherapy. We demonstrated that, in carefully selected cases, such as those with substantial tumor downstaging, controlled metastatic disease with residual intrathoracic involvement, or isolated thoracic relapse, surgical resection can be safely performed with excellent perioperative outcomes. In a landscape where over 60% of NSCLC cases are diagnosed at a locally advanced or metastatic stage with poor prognosis, salvage surgery should no longer be viewed merely as a last-resort intervention. When integrated into a multidisciplinary treatment strategy, it may offer a meaningful survival benefit and a real chance of cure in carefully selected patients responding to immunotherapy-based regimens.

## 1. Introduction

In recent years, a multidisciplinary approach combining chemotherapy, immunotherapy, and radiotherapy has opened new therapeutic possibilities for patients with advanced-stage lung cancer. This integrated strategy has significantly expanded treatment options and improved outcomes [1].

Concurrent chemoradiotherapy followed by consolidation immunotherapy with durvalumab, as validated by the PACIFIC trial, currently represents the standard of care for unresectable stage III non-small cell lung cancer (NSCLC) [1]. However, in real-world practice, treatment strategies are often more heterogeneous, especially in patients with advanced or metastatic disease, where radiotherapy is sometimes directed to metastatic sites rather than to the primary tumor. This heterogeneity reflects the complexity of patient presentation and multidisciplinary decision-making in clinical settings outside randomized trials.

Chemo-immunotherapy has been introduced as a first-line treatment for advanced NSCLC, aiming to reduce tumor burden and improve prognosis. In selected cases, the tumor regression may prompt reconsideration of surgical resection [2].

The term salvage surgery refers to surgical intervention performed after definitive chemotherapy, immunotherapy, or chemoradiotherapy in patients with NSCLC initially deemed unresectable, due to insufficient treatment response, recurrence, or complications rendering surgery the only curative option [3]. Salvage surgery is also considered for patients with locally advanced NSCLC (stage IIIA–IIIB) who, after neoadjuvant therapy (chemotherapy, chemoradiotherapy, or chemo-immunotherapy), develop complications or fail to respond adequately and thus require prompt surgical management [4]. Recent studies have evaluated the feasibility and outcomes of salvage surgery across various clinical scenarios. The growing application of this approach is partly attributable to advances in immunotherapy, which has become a cornerstone in lung cancer treatment. In particular, immune checkpoint inhibitors (ICIs) have revolutionized therapeutic strategies by significantly improving survival and quality of life, even in advanced disease stages. Consequently, new curative indications for salvage surgery are emerging in patients with locally advanced NSCLC initially considered unresectable, but who achieve substantial responses to ICIs [5,6].

Although preliminary results in terms of pathological response are promising, the associated surgical risks and perioperative morbidity have not been fully characterized. Several technical challenges and potential perioperative risks have been reported, primarily related to dense adhesions, hilar inflammation, and fibrosis of perivascular structures secondary to hyperactivation of the inflammatory response [7].

Nonetheless, in light of recent findings, salvage surgery is gaining recognition as a safe and effective therapeutic option for carefully selected patients with initially unresectable NSCLC, expanding treatment possibilities within a multidisciplinary framework [8].

The aim of this study was to evaluate the feasibility, safety, and efficacy of salvage surgery following chemo-immunotherapy in patients with locally advanced NSCLC initially considered inoperable due to anatomical or biological tumor characteristics.

## 2. Materials and Methods

### 2.1. Study Design and Patient Selection

We conducted a retrospective analysis of prospectively collected data on all patients diagnosed with initially unresectable NSCLC who underwent definitive chemo-immunotherapy and were subsequently considered for salvage surgery between January 2019 and June 2024 at our tertiary care center.

At diagnosis, all patients underwent comprehensive imaging, including contrast-enhanced total-body computed tomography (CT), 18 F-fluorodeoxyglucose positron emission tomography (18 F-FDG PET), and brain magnetic resonance imaging (MRI) when necessary for staging. Flexible bronchoscopy with or without endobronchial ultrasound (EBUS) was performed in cases with radiological suspicion of airway or mediastinal lymph node involvement, in accordance with European Society of Thoracic Surgeons (ESTS) guidelines [9]. Histological confirmation was obtained via CT-guided percutaneous needle biopsy when endoscopic methods were non-diagnostic. Molecular profiling was then conducted to assess PD-L1 expression and detect actionable mutations, enabling tailored systemic therapy.

Clinical staging was based on the 8th edition of the TNM classification system [10]. Patients included in this study were initially deemed inoperable by the Multidisciplinary Tumor Board (MTB) and underwent definitive chemo-immunotherapy. Patients with wild-type NSCLC and high PD-L1 expression received platinum-based chemotherapy (cisplatin or carboplatin) in combination with paclitaxel, nab-paclitaxel, or pemetrexed, plus an ICI (pembrolizumab or atezolizumab). For patients with HER2-mutant NSCLC, treatment included carboplatin and paclitaxel combined with trastuzumab. In patients with brain or bone metastases, stereotactic body radiation therapy (SBRT) was delivered to the metastatic sites, in addition to systemic treatment.

After at least two cycles of therapy, treatment response was re-evaluated by the MTB using clinical, laboratory, and imaging criteria. Patients deemed potentially resectable with radical intent were considered for salvage surgery.

Surgery was recommended according to the following criteria:

Incomplete or poor response to chemo-immunotherapy, with complications requiring surgical intervention;

Substantial clinical response allowing resection of the primary tumor;

Good control of distant metastases, with residual intrathoracic disease;

Isolated intrathoracic recurrence after completion of systemic treatment.

In addition to these general criteria, specific technical aspects were considered during restaging. Patients with persistent bulky mediastinal disease, extensive vascular invasion, or central tumors judged unresectable on CT scan or bronchoscopy/EBUS were excluded. Surgical candidacy required the possibility of achieving a complete (R0) resection based on radiological and endoscopic assessment, together with adequate cardiopulmonary reserve.

### 2.2. Pre-Operative Work-Up and Surgical Procedure

Preoperative restaging was performed within 30 days prior to surgery and included contrast-enhanced total-body CT, 18 F-FDG PET, and brain MRI when indicated. A comprehensive evaluation of cardiopulmonary function was also conducted.

All patients underwent lung resection with radical intent, primarily through anatomical resections (lobectomy, bilobectomy, or pneumonectomy). When anatomical resection was not feasible due to post-treatment fibrosis or lymph node adhesions to broncho-vascular structures, non-anatomical pulmonary resection (wedge) was performed.

In most cases, an open approach (posterolateral thoracotomy) was employed, extended via the Shaw–Paulson technique in apical tumors. Selected patients underwent uniportal video-assisted thoracoscopic surgery (VATS).

When mediastinal or chest wall structures were involved, en bloc resection was performed (including pericardium, aortic adventitia, or ribs as needed). Frozen section analysis was used intraoperatively to assess margin status when tumor involvement was suspected. Systematic lymphadenectomy was performed according to international guidelines, or, when not feasible, sampling of accessible nodal stations was performed [11].

### 2.3. Histopathological Evaluation

Pathological response was assessed according to the recommendations of the International Association for the Study of Lung Cancer (IASLC) [12]. Residual viable tumor was quantified by estimating the cross-sectional area of viable tumor versus areas of necrosis and fibrosis (tumor bed) in each section. The mean percentage of viable tumor cells per patient was calculated. A major pathological response (MPR) was defined as <10% viable tumor cells, and a complete pathological response (pCR) was defined as no viable tumor cells. The same criteria were applied to evaluate the effect of treatment on metastatic lymph nodes.

### 2.4. Post-Operative Management and Data Collection

After surgery and histopathological evaluation, patients were reassessed by the MTB and were either referred to maintenance immunotherapy or, in cases of persistent nodal involvement, to adjuvant chemo-immunotherapy combined with radiotherapy. In cases of pCR after prolonged treatment or in the presence of drug-induced toxicity, patients were referred to exclusive follow-up.

Radiological follow-up included contrast-enhanced CT and/or 18 F-FDG PET scans every 4 months for the first 2 years, every 6 months for the following 3 years, and annually thereafter, based on oncological recommendations.

Collected variables included demographic and clinical data (age, sex, smoking history, body mass index [BMI], performance status, comorbidities); oncologic variables (clinical stage at diagnosis, histology, molecular profile, PD-L1 expression, initial reason of inoperability, treatment regimen and cycles, associated radiotherapy, adverse effects, adjuvant therapy); surgical data (type and extent of resection, approach, operative time, radicality). Intra- and post-operative mortality and morbidity were recorded. Complications were classified using the Common Terminology Criteria for Adverse Events (CTCAE), version 5.0 [13]. Oncologic outcomes were also assessed, including pathological stage, complete (R0) resection rate, pCR, MPR, recurrence, progression-free survival (PFS), and overall survival (OS).

The study was conducted according to the guidelines of the Declaration of Helsinki and approved by the Institutional Review Board CEAVNO (IRB approval number N. 21906/16.02.2023). Informed consent was obtained from all subjects involved in the study before data collection, and its use in clinical studies was made in an anonymous form. Moreover, they gave their consent for the publication of their data and to update the database information. The STROBE reporting recommendations were used in the reporting of the present study [14]. The STROBE checklist is available as the Supplemental File S1.

### 2.5. Statistical Analysis

Continuous variables were tested for normal distribution by the Shapiro–Wilk test and were expressed as mean and standard deviation (SD) if normally distributed, or as median and interquartile range (IQR) if not normally distributed. Categorical variables were expressed as absolute numbers and percentages.

The primary end-points were PFS and OS. The secondary end-points assessed were pathological responses (pCR and MPR) and postoperative complications rates. PFS was defined as the time from surgery to the first recurrence or last follow-up (December 2024). OS was defined as the time from surgery to death from any cause or last follow-up. Survival outcomes were estimated using the Kaplan–Meier method with 95% confidence intervals (CIs) and compared using the log-rank test.

Prognostic factors for OS and PFS were evaluated using Cox proportional hazards regression models. The statistical significance level was set as a *p*-value < 0.05. The proportional hazards assumption was tested using log(-log) survival plots. Multivariable analyses were conducted, including only variables that reached statistical significance in the univariate analyses. The variables tested in the univariate analyses were selected based on clinical and oncological relevance and comprised age, sex, smoking status, BMI, tumor histology, tumor stage, type of surgical resection, and pathological response. A minimum of approximately ten events per covariate was required to assess prognostic factors for OS and PFS.

Statistical analyses were conducted using SPSS software (Statistical Package for the Social Sciences, version 23.0; IBM Corp., Armonk, NY, USA).

## 3. Results

### 3.1. Patients

A total of 21 patients were enrolled in this study, including 13 males (61.9%), with a median age of 68 years (IQR: 9) and a mean BMI of 25.9 (4.6) kg/m^2^. Most patients were current or former smokers (19 cases, 90.5%) and had a history of cardiovascular disease (13 cases, 61.9%). The majority of patients had good performance status, with an Eastern Cooperative Oncology Group Performance Status (ECOG-PS) score of 0 in 18 cases (85.7%) [15].

The most common histological subtype was adenocarcinoma, observed in 14 patients (66.7%), followed by squamous cell carcinoma in 5 (23.8%) and other histology in 2 (9.5%). At diagnosis, stage IIIA was the most frequent (10 cases, 47.6%), followed by stages IVA (6 patients, 28.6%), IIIB (4 patients, 19%), and IVB (1 patient, 4.8%).

Criteria for initial unresectability included local invasion in seven patients (33.3%), distant metastases in six (28.6%), bulky N2 disease in three (14.3%), and multiple unresectability criteria in five patients (23.8%).

The mean PD-L1 expression in the study population was 22% (30%). All patients underwent definitive chemo-immunotherapy, with a median of four cycles (IQR: 1). The most frequently administered ICI was pembrolizumab (16 cases, 76%). In one patient (4.8%), grade 3 toxicity occurred after two cycles of monalizumab and durvalumab, leading to treatment discontinuation. Radiotherapy was administered as part of the treatment for the primary lung tumor in three patients (14.3%).

Among the seven patients with stage IV NSCLC, metastatic sites were treated with surgery in two cases (28.6%), radiotherapy in four cases (57.1%), and a combination of both in one case (14.3%).

Following at least two cycles of chemo-immunotherapy, treatment response was assessed by the MTB and patients with the subsequent criteria were considered eligible for salvage surgery: incomplete or poor treatment response with complications requiring surgical management (four patients, 19%); substantial clinical response allowing resection of the primary tumor (eight patients, 38.1%); good control of distant metastases with residual intrathoracic disease (six patients, 28.6%); or an isolated intrathoracic recurrence occurring after completion of systemic therapy (three patients, 14.3%).

The main demographic and clinical characteristics are summarized in Table 1.

### 3.2. Surgical and Postoperative Outcomes

The median time from therapy to surgery was 45 days (IQR: 33). A uniportal VATS approach was feasible in 2 patients (9.5%) without the need for conversion, while a posterolateral thoracotomy was performed in the remaining 19 patients (90.5%). The mean operative time was 189.8 (41.9) minutes.

Lung resections included 14 lobectomies (66.7%), 4 wedge resections (19%), 2 left pneumonectomies (9.5%), and 1 lower bilobectomy (4.8%). Extended resections were required in eight patients (38.1%), including three lobectomies extended to adjacent lobar segments, three enbloc chest wall resections involving at least two ribs, one pericardial resection, and one resection of the adventitia of the descending thoracic aorta.

No intraoperative complications were reported. Postoperative complications occurred in nine patients (43%). Five patients (55.6%) experienced Grade 2 complications: one case of chylothorax managed with a fat-free diet, one case of prolonged air leak managed conservatively, and three cases of moderate anemia treated with blood transfusion. Three patients (33.3%) developed Grade 3 complications: one pleural empyema managed with chest drainage and antibiotic therapy, and two cases of severe anemia requiring transfusion and iron supplementation. One patient (11.1%) experienced a Grade 1 complication: mild anemia treated with iron therapy alone.

When analyzing the relationship between the extent of resection and postoperative morbidity, we observed that neither of the two pneumonectomies nor the single bilobectomywas associated with major postoperative complications (≥grade 4). No correlation between the surgical extent and severity of complications was identified in this series (*p* = 0.207).

The median length of hospital stay was 7 days (IQR: 4). No intraoperative or 30-day mortality occurred. One patient (4.8%) died within 90 days due to rapid disease progression in the context of immunosuppression.

### 3.3. Oncological Outcomes

The complete (R0) resection rate was 100% (21 patients). pCR was achieved in seven cases (33.3%) and MPR in three cases (14.3%). At pathologic staging, seven patients (33.3%) had no evidence of disease, six (28.6%) were classified as stage IIA or IIB, four (19.0%) as stage IIIA or IIIB, three (14.3%) as stage IA or IB, and one patient (4.8%) as stage IVA. Overall, tumor downstaging was observed in 16 patients (76.2%), while nodal downstaging occurred in 7 cases (33.3%) and nodal upstaging in 2 cases (9.5%).

Postoperatively, 12 patients (57.1%) received maintenance immunotherapy alone (1 volrustomig, 1 atezolizumab, 10 pembrolizumab); 4 patients (19.0%) underwent chemo-immunotherapy (2 pemetrexed/pembrolizumab, 1 carboplatin–paclitaxel/trastuzumab, 1 carboplatin–pemetrexed/pembrolizumab), and 1 patient (4.8%) received adjuvant chemotherapy with cisplatin/pemetrexed only, due to a previous immune-related adverse event. Adjuvant radiotherapy was administered in four patients (19.0%) with mediastinal nodal involvement.

After a median follow-up of 17 months (IQR: 19), there were five deaths (23.8%), four due to disease progression, while 14 patients (66.6%) were alive and disease-free at the last evaluation. During follow-up, seven patients (33.3%) developed disease recurrence, managed with radiotherapy or chemo(immuno)therapy depending on site and extent. Specifically, one patient (4.8%) experienced isolated locoregional recurrence, three (14.3%) developed distant metastases (adrenal gland, brain, bone, and liver), and three (14.3%) had both locoregional and distant recurrences.

**Figure 1:**
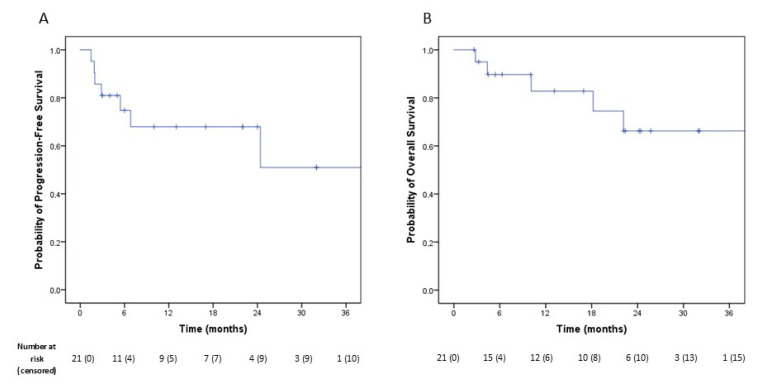
(**A**): Kaplan–Meier curve showing cumulative progression-free survival of the whole cohort. (**B**): Kaplan–Meier curve showing cumulative overall survival of the whole cohort.

The estimated 3-year PFS and OS rates were 50.9% and 66.3%, respectively (Figure 1).

**Figure 2:**
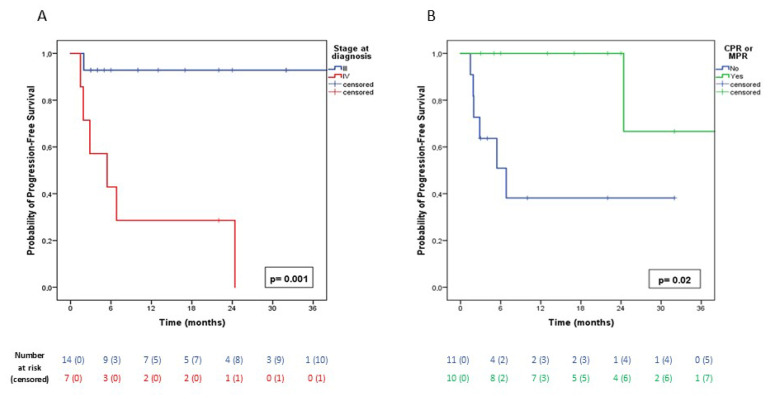
(**A**): Kaplan–Meier curves showing cumulative progression-free survival (PFS) in patients with stage III NSCLC at diagnosis (blue line) versus patients with stage IV NSCLC (red line). (**B**): Kaplan–Meier curves showing cumulative PFS in patients who achieved pCR or MPR (green line) versus those who did not (blue line).

The mean cumulative PFS, estimated by Kaplan–Meier analysis, was 41.0 months (SE: 9.0; 95% CI: 23.3–58.7). Patients with higher clinical stage at diagnosis and those without pCR or MPR had significantly worse PFS. Specifically, mean PFS for stage III versus stage IV patients was 65.1 months (SE: 4.7; 95% CI: 55.9–74.3) vs. 9.6 months (SE: 3.9; 95% CI: 1.8–17.3), *p* = 0.001 (Figure 2A); mean PFS for patients achieving pCR or MPR was 54.8 months (SE: 12.4; 95% CI: 30.5–79.1) compared with 14.5 months (SE: 4.6; 95% CI: 5.5–23.5) in those without, *p* = 0.020 (Figure 2B).

**Figure 3:**
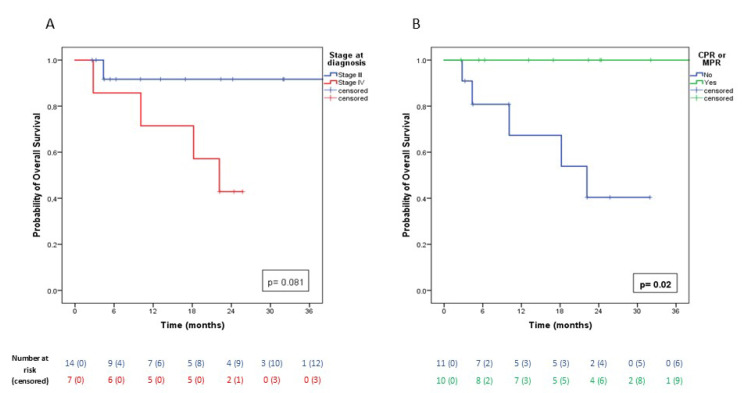
(**A**): Kaplan–Meier curves showing cumulative overall survival (OS) in patients with stage III NSCLC at diagnosis (blue line) versus patients with stage IV NSCLC (red line). (**B**): Kaplan–Meier curves showing cumulative OS in patients who achieved pCR or MPR (green line) versus those who did not (blue line).

Mean OS was 51.0 months (SE: 7.2; 95% CI: 36.8–65.0). OS analysis showed no statistically significant difference according to clinical stage at diagnosis (*p* = 0.081), although survival curves demonstrated a marked reduction in OS for stage IV patients (Figure 3A). In contrast, achieving pCR or MPR after chemo-immunotherapy was a significant prognostic factor for OS (*p* = 0.020), with markedly worse survival in patients without such responses (Figure 3B).

On univariate Cox regression analysis, no variables were identified as significant predictors of OS. Conversely, clinical stage III at diagnosis (OR: 0.257; 95% CI: 0.089–0.743; *p* = 0.012) and achievement of pCR or MPR (OR: 0.113; 95% CI: 0.013–0.959; *p* = 0.046) were significantly associated with reduced recurrence risk. Among the ten patients with pCR or MPR, only one developed a distant relapse two years after surgery, namely an isolated adrenal metastasis treated with radiotherapy. At the last follow-up, all 10 were alive and disease-free.

Multivariable analysis confirmed lower clinical stage at diagnosis (stage III vs. stage IV) as the single independent positive predictor of PFS (OR: 0.292; 95% CI: 0.093–0.912; *p* = 0.034). The results of the Cox regression analyses are summarized in Table 2 and Table 3.

## 4. Discussion

Salvage surgery has traditionally been reserved as the ultimate therapeutic option for patients with advanced, unresectable NSCLC who develop complications such as lack of response to definitive oncologic therapy or isolated locoregional relapse [3]. In these situations, surgery was viewed as the last remaining opportunity to improve prognosis once systemic treatment (chemoradiotherapy and/or immunotherapy) was no longer suitable [4]. Consequently, it was regarded as a “last-chance” intervention, performed with palliative or exceptional intent rather than as a standard curative strategy.

The introduction of ICIs has significantly changed this perspective. Salvage surgery is increasingly recognized as feasible and potentially curative in carefully selected patients with locally advanced NSCLC initially considered inoperable but achieving major tumor regression and durable disease control after immunotherapy-based treatment [5,6]. Over the past decade, the use of multidisciplinary approaches, combining chemotherapy, immunotherapy, and, when appropriate, radiotherapy, has broadened the population reconsidered for resection after initial unresectability [1,2,3]. Several landmark and ongoing trials (NADIM, PACIFIC, IMpower-010, KEYNOTE-091, NeoCOAST2, among others) support the integration of ICIs into neoadjuvant, adjuvant, and definitive regimens [1,16,17,18,19]. ICIs have shown strong efficacy in advanced NSCLC, leading to significant survival and quality of life gains [20,21]. Radiotherapy, either as conventional fractionation or SBRT, remains crucial in advanced disease. Beyond symptom control, it can reduce tumor volume, enhance systemic therapy effectiveness, and improve resectability. When combined with chemo-immunotherapy, it exerts a synergistic effect that increases the likelihood of a surgical window [2].

It should be emphasized that, although concurrent chemoradiotherapy followed by durvalumab is the established standard of care for unresectable stage III NSCLC [1], only a minority of patients in our series received radiotherapy to the primary lung tumor. In other cases, radiotherapy was delivered to metastatic sites such as the brain or bone. This treatment heterogeneity reflects real-world multidisciplinary practice, where systemic therapy is prioritized in advanced disease, and radiotherapy is tailored to disease burden and location.

In selected patients, the tumor response to ICIs has the potential to transform salvage surgery from an unplanned, reactive procedure into an intentional component of curative-intent therapy. This might represent a conceptual shift: rather than intervening only when other treatments fail, salvage surgery may now be proactively considered in the treatment algorithm for patients with substantial tumor downstaging, effective control of distant metastases with residual intrathoracic disease, or isolated thoracic relapse after systemic therapy. In optimal scenarios, this integration can yield prolonged survival and, in a subset of patients, complete remission.

**Figure 4:**
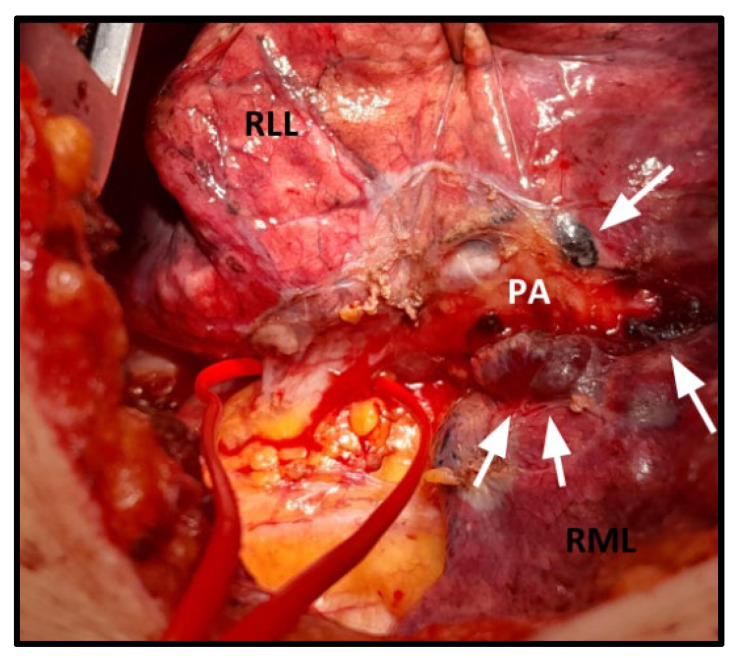
Intraoperative photo showing lymph nodes (white arrows) with fibrosis and adhesions to the right pulmonary artery after chemo-immunotherapy. RLL: right lower lobe; PA: pulmonary artery; RML: right middle lobe.

However, this evolving role involves specific technical and perioperative challenges. ICIs trigger an intense immune-mediated inflammatory cascade, often replacing tumor tissue with necrosis and/or dense fibrosis [22,23,24]. These changes, frequently accompanied by adhesions and peribronchovascular remodeling, complicate essential maneuvers such as vessel isolation, bronchial dissection, and mediastinal lymphadenectomy (Figure 4). Such complexity may prolong surgery and increase intraoperative risks, especially during minimally invasive procedures where conversion rates may be higher [7]. Chaft et al. first described marked fibrosis in hilar and mediastinal lymph nodes after ICIs, noting that it hindered standard dissection [25].

Our findings confirm that, when performed in high-volume centers by experienced thoracic surgeons, salvage surgery after definitive chemo-immunotherapy is feasible and safe, with negligible morbidity. In our cohort, complete (R0) resection was achieved in all patients. Most procedures (90.5%) were performed via open thoracotomy, reflecting post-immunotherapy tissue complexity, while two cases (9.5%) were completed by uniportal VATS without conversion. These results align with the NADIM trial, where complete resection reached 89% and conversion occurred in 19%, reaffirming the technical challenges posed by fibrosis and adherent lymphadenopathy [7,17,25].

In our series, four patients (19%) were unable to undergo anatomical resection due to extensive peribronchovascular tissue remodeling or necrotic/colliquative lymphadenopathy involving hilar or interbronchial regions, requiring atypical sublobar resections. Notably, this did not negatively influence OS (*p* = 0.73) or PFS (*p* = 0.66), suggesting that in carefully selected cases, non-anatomical resections may still yield favorable oncologic outcomes when R0 margins are obtained.

Perioperative safety outcomes in our study were highly satisfactory. There were no intraoperative deaths or major intraoperative complications. Thirty-day mortality was zero, and 90-day mortality was 4.8%, related to rapid disease progression in an immunocompromised patient. Postoperative complications occurred in 43% of patients, all graded ≤ 3 on the CTCAE scale [13]. The most frequent were prolonged air leak and postoperative anemia, both managed conservatively without reoperation.

The role of pneumonectomy in the context of salvage surgery after chemo-immunotherapy deserves specific consideration. Historically, pneumonectomy following induction therapy has been associated with high morbidity and mortality, particularly due to technical complexity and impaired postoperative tolerance. In our cohort, two patients underwent left pneumonectomy after significant tumor response, both without intraoperative complications or perioperative mortality. Although limited by small numbers, these results suggest that pneumonectomy may be safely performed in highly selected patients when required to achieve complete resection.

The potential perioperative complications, together with the specific technical difficulties related to exclusive chemo-immunotherapy, highlight the need for careful and appropriate patient selection by a multidisciplinary team when considering salvage surgery. The satisfactory safety profile and favorable postoperative outcomes observed in our series should therefore be interpreted in light of a rigorous selection process that considers treatment response, comorbidities, and disease extent, as well as the performance of the procedure by highly experienced surgeons in specialized, high-volume centers.

Comparable results after salvage surgery following immunotherapy for advanced NSCLC have been documented in the literature. Ueno et al. reported morbidity and perioperative mortality rates of 27% and 0%, respectively, with a 90-day mortality of 9% [26]. Schiavon et al. found postoperative complications in 25% of cases and no perioperative mortality [27], while Bott et al. and Guerrera et al. reported minor perioperative complication rates of approximately 30% [5,7]. In contrast, Higuchi et al. observed a 30.8% complication rate, mostly grade ≥ 3, underscoring variability across institutions and patient cohorts [28].

Oncological outcomes were also encouraging in our series, with pCR observed in 33.3% and MPR in 14.3% of patients. Literature consistently associates ICI-based regimens with higher pCR and MPR rates compared to chemotherapy alone. In the neoadjuvant setting, the CHECKMATE 816 trial reported pCR in 30.5% [22], and Cao et al. documented pCR in 24% of primary tumors and 20% of lymph nodes [29]. Achieving pCR or MPR in our cohort correlated with better PFS (54.8 vs. 14.5 months, *p* = 0.02) and OS (57.1 vs. 22.2 months, *p* = 0.02), and with lower recurrence risk (OR: 0.113; 95% CI: 0.013–0.959; *p* = 0.046). Among the ten patients with pCR/MPR, only one relapsed (a solitary adrenal metastasis treated with radiotherapy); all were alive and disease-free at the last follow-up. Our pCR/MPR results were slightly superior to those of Higuchi et al. [28] (pCR 30.8%) and Guerrera et al. [7] (pCR 31%, with 7% residual N2 disease), and slightly lower than those of Schiavon et al. [27] (pCR 48%, MPR 16%), possibly reflecting differences in sample size and patient selection criteria. Regardless, the consistent association between significant pathological response and improved survival reinforces the prognostic significance of pCR/MPR after chemo-immunotherapy.

Although direct comparison with neoadjuvant trials is not feasible, real-world data on salvage surgery after definitive chemo-immunotherapy appear highly promising, showing encouraging results in terms of both pathological response and medium- to long-term oncological outcomes [30,31,32,33]. In our study, 3-year PFS and OS rates were 50.9% and 66.3%, respectively, despite all patients being initially unresectable, a population usually associated with poor prognosis. These outcomes are comparable to those reported by Hamaji et al. [33] (3-year PFS 57.9%, OS 68.2%) and Guerrera et al. [6] (3-year PFS 60%, OS 79%). Considering the limitations posed by varying follow-up durations, Higuchi et al. reported a 2-year OS of 76.2% after salvage surgery for advanced NSCLC following ICIs [28], which was higher in patients without complications and with post-treatment stage 0, compared to those with stage II disease and severe complications. Similarly, Bott et al. reported a 2-year OS of 77% [5]. These findings suggest that salvage surgery, within a multimodal treatment strategy, can achieve survival outcomes approaching those of patients resected at earlier stages.

This study has several limitations. Its retrospective design and small sample size, combined with the absence of a control group, limit the generalizability of the results. Moreover, heterogeneity in preoperative treatment regimens, including variations in chemotherapy protocols, types of ICIs, and radiotherapy, precludes direct comparisons. Finally, although the follow-up duration was adequate to evaluate mid-term outcomes, longer follow-up is required to assess survival, particularly in patients with stage IV disease.

Given the promising real-world results, future research should focus on prospective, multicenter trials with standardized criteria and protocols to better assess the safety, oncological effectiveness, and long-term benefits of salvage surgery after definitive chemo-immunotherapy. These studies should also incorporate translational end-points such as biomarker analyses to identify patients most likely to benefit, alongside evaluations of quality of life, functional recovery, and cost-effectiveness to optimize the integration of salvage surgery into NSCLC treatment algorithms.

## 5. Conclusions

This paper is an extended version of our study recently presented [34].

Our findings confirm that salvage surgery after definitive chemo-immunotherapy in initially unresectable NSCLC is feasible, safe, and effective when performed in specialized, high-volume centers. Complete resection was achieved in all patients, perioperative morbidity was acceptable, and mortality was minimal. A significant pathological response (pCR or MPR) was strongly associated with improved survival, underscoring its prognostic value.

Salvage surgery, once considered only a last resort, is emerging as a proactive, potentially curative option for carefully selected NSCLC patients with tumor downstaging, durable metastatic control, or isolated thoracic recurrence. Integrating this approach within a multidisciplinary strategy could redefine treatment algorithms, and prospective, multicenter research is essential to validate its long-term benefits.

## Figures and Tables

**Table 1:** Demographic and clinical characteristics of the study population.

Variable	Description
**Sex** (*n*, %)	
M	13 (61.9)
F	8 (38.1)
**Median age** * (years, IQR)	68 (9)
**Smoking status** (*n*, %)	
Current smokers	10 (47.6)
Former smokers	9 (42.9)
Never	2 (9.5)
**BMI** (*n*, %)	
<25	8 (38.1)
25–30	9 (42.9)
>30	4 (19)
**Comorbidity** (*n*, %)	
Cardiovascular	13 (61.9)
Previous malignancy	6 (28.6)
Diabetes Mellitus	5 (23.8)
Respiratory	4 (19)
COPD	2 (9.5)
OSAS	1 (4.8)
Pulmonary fibrosis	1 (4.8)
Gastrointestinal	3 (14.3)
**ECOG-PS Score** (*n*, %)	
0	18 (85.7)
1	3 (14.3)
**Preoperative pulmonary function test** (mean, SD)	
FEV1 (%)	86 (12)
FVC (%)	88 (11)
DLCO (%)	76 (8)
**PD-L1** (*n*, %)	
<50%	17 (81)
≥50%	4 (19)
**Primary tumor location** (*n*, %)	
RUL	3 (14.3)
RML	1 (4.8)
RLL	5 (23.8)
LUL	9 (42.8)
LLL	3 (14.3)
**Metastasis location** (*n*, %) Contralateral lung Brain Bone	(N = 7 patients) 3 (43) 3 (43) 1 (14)
**Immunotherapy drugs associated with traditional chemotherapy** (*n*, %)	
Pembrolizumab	16 (76)
Trastuzumab	1 (4.8)
Atezolizumab	1 (4.8)
Durvalumab	1 (4.8)
Monalizumab/Durvalumab	1 (4.8)
Volrustomig	1 (4.8)
**Cycles of immunotherapy associated with traditional chemotherapy** (*n*, %)	
2	2 (9.5)
3	3 (14.3)
4	7 (33.3)
6	4 (19)
7	1 (4.8)
8	1 (4.8)
10	2 (9.5)
31	1 (4.8)
**Site of radiotherapy** (*n*, %)	
Brain	3 (14.3)
Bone (iliac lesion)	1 (4.8)
Primitive lung tumor	3 (14.3)

* Continuous variable. IQR: Interquartile Range; BMI: Body Mass Index; COPD: Chronic Obstructive Pulmonary Disease; OSAS: Obstructive Sleep Apnea Syndrome; ECOG-PS: Eastern Oncology Cooperative Group Performance Status; SD: standard deviation; FEV1: Forced Expiratory Volume in 1 s; FVC: Forced Vital Capacity; DLCO: Diffusing capacity for carbon monoxide; PD-L1: Programmed Death–Ligand 1; RUL: Right Upper Lobe; RML: Right Middle Lobe; RLL: Right Lower Lobe; LUL: Left Upper Lobe; LLL: Left Lower Lobe.

**Table 2:** Univariate Cox regression analysis for overall survival.

**Variable**	**OR (95% C.I.)**	***p*** **-Value**
Age *	1.013 (0.904–1.134)	0.83
Male sex	43.690 (0.023–84068.084)	0.33
Former or current smokers	0.211 (0.019–2.345)	0.20
BMI *	0.956 (0.750–1.219)	0.72
Adenocarcinoma	1.547 (0.515–4.647)	0.44
Clinical stage III at diagnosis	0.418 (0.139–1.258)	0.12
Anatomical lung resection	0.826 (0.274–2.490)	0.73
Major or Complete Pathological Response	8.283 (0.226–303.978)	0.25

OR: Odds Ratio; C.I.: Confidence Interval; BMI= Body Mass Index. * Continuous variable.

**Table 3:** Univariate and multivariable Cox regression analyses for progression-free survival.

	Univariate Analysis		Multivariable Analysis	
**Variable**	**OR (95% C.I.)**	***p*-Value**	**OR (95% C.I.)**	***p*-Value**
Age *	1.023 (0.939–1.114)	0.61		
Male sex	58.638 (0.127–27096.376)	0.19		
Former or current smoker	0.199 (0.020–1.954)	0.17		
BMI *	0.887 (0.716–7.099)	0.27		
Adenocarcinoma	1.971 (0.681–5.707)	0.21		
**Clinical stage III at diagnosis**	**0.257 (0.089–0.743)**	**0.012**	**0.292 (0.093–0.912)**	**0.034**
Anatomical lung resection	1.265 (0.437–3.658)	0.66		
**Major or Complete Pathological Response**	**0.113 (0.013–0.959)**	**0.046**	0.140 (0.010–2.038)	0.150

OR: Odds Ratio; C.I.: Confidence Interval; BMI= Body Mass Index. * Continuous variable. Bold values indicate statistical significance.

## Data Availability

The article’s underlying data will be shared upon reasonable request to the corresponding author.

## Supplementary Materials

The following supporting information can be downloaded at: https://www.mdpi.com/article/10.3390/cancers17182967/s1, Supplemental File S1: STROBE checklist.

## Abbreviations

The following abbreviations are used in this manuscript:

NSCLC	Non-Small Cell Lung Cancer
IQR	Interquartile range
ICIs	Immune Checkpoint Inhibitors
pCR	Complete Pathological Response
MPR	Major Pathological Response
PFS	Progression-Free Survival
OS	Overall Survival
CT	Computed Tomography
18 F FDG PET	18 fluorodeoxyglucose positron emission tomography
MRI	Magnetic Resonance Imaging
EBUS	Endobronchial ultrasound
ESTS	European Society of Thoracic Surgery
PD-L1	Programmed Cell Death Ligand 1
TNM	Tumor Node Metastasis
MTB	Multidisciplinary Tumor Board
HER 2 SBRT	Human epidermal growth factor receptor 2 Stereotactic body radiation therapy
VATS	Video-assisted thoracoscopic surgery
IASCL	International Association for the Study of Lung Cancer
BMI	Body Mass Index
CTCAE	Common Terminology Criteria for Adverse Events
CEAVNO	Comitato Etico Regione Toscana-Area Vasta Nord Ovest
SPSS	Statistical Package for the Social Sciences
SD	Standard Deviation
ECOG-PS COPD OSAS FEV1 FVC DLCO	Eastern Cooperative Oncology Group Performance Status Chronic Obstructive Pulmonary Disease Obstructive Sleep Apnea Syndrome Forced Expiratory Volume in 1 s Forced Vital Capacity Diffusing capacity for carbon monoxide
OR	Odd Ratio
SE	Standard error
CI	Confidence Interval
RUL	Right Upper Lobe
RLL	Right Lower Lobe
RML	Right Middle Lobe
LUL	Left Upper Lobe
LLL	Left Lower Lobe
